# Oxidative Stress Related Diseases in Newborns

**DOI:** 10.1155/2016/2768365

**Published:** 2016-06-15

**Authors:** Yasemin Ozsurekci, Kubra Aykac

**Affiliations:** Department of Pediatric Infectious Diseases, Hacettepe University, Faculty of Medicine, 06100 Ankara, Turkey

## Abstract

We review oxidative stress-related newborn disease and the mechanism of oxidative damage. In addition, we outline diagnostic and therapeutic strategies and future directions. Many reports have defined oxidative stress as an imbalance between an enhanced reactive oxygen/nitrogen species and the lack of protective ability of antioxidants. From that point of view, free radical-induced damage caused by oxidative stress seems to be a probable contributing factor to the pathogenesis of many newborn diseases, such as respiratory distress syndrome, bronchopulmonary dysplasia, periventricular leukomalacia, necrotizing enterocolitis, patent ductus arteriosus, and retinopathy of prematurity. We share the hope that the new understanding of the concept of oxidative stress and its relation to newborn diseases that has been made possible by new diagnostic techniques will throw light on the treatment of those diseases.

## 1. Introduction

*(1) Oxidative Stress.* There is a crucial balance between free radical production and antioxidant defense mechanisms. While human bodies are producing energy, molecules with one or more unpaired electrons in their outer shell, called free radicals, occur in the respiratory chain, phagocytosis, prostaglandin synthesis, and the cytochrome P450 system [1–3]. Free radicals are formed from molecules via the breaking of a chemical bond such that each fragment keeps one electron, via cleavage of radicals to give other radicals and via redox reactions [3, 4]. Current known free radicals are hydroxyl (OH•), superoxide (O_2_^−^•), nitric oxide (NO•), nitrogen dioxide (NO_2_•), peroxyl (ROO•), and lipid peroxyl (LOO•). In addition, hydrogen peroxide (H_2_O_2_), ozone (O_3_), singlet oxygen (^1^O_2_), hypochlorous acid (HOCl), nitrous acid (HNO_2_), peroxynitrite (ONOO^−^), dinitrogen trioxide (N_2_O_3_), and lipid peroxide are not free radicals but are called oxidants, because they can easily lead to free radical reactions in organisms [5]. Reactive oxygen species (ROS) include both free radicals and nonfree radical oxygenated molecules. Reactive nitrogen, iron, copper, and sulfur species are also encountered. Oxidative stress and imbalance of the redox reaction can be originated by those radical species. ROS/reactive nitrogen species- (RNS-) induced damage in oxidative stress is considered a contributing factor to the pathogenesis and pathophysiology of many health problems, either as a source or as an outcome [1, 6, 7]. Overexpression of oncogenes and generation of mutagen compounds or inflammation leads to some diseases such as cancer, and neurodegeneration may be affected by the involvement of ROS/RNS species [1, 5, 8, 9].

ROS and RNS are generated from either endogenous or exogenous sources. Endogenous free radicals are produced from immune cell activation, inflammation, mental stress, excessive exercise, ischemia, infection, cancer, and aging. Exogenous ROS/RNS is caused by air and water pollution; cigarette smoke; alcohol; heavy or transition metals; certain drugs including cyclosporine, tacrolimus, gentamycin, and bleomycin; industrial solvents such as asbestos; cooking (smoked meat, used oil, and fat); and radiation. After penetration into the body by different routes, these exogenous compounds are decomposed or metabolized into free radicals [6, 10–15].

However, free radicals are not always harmful; at low or moderate concentrations, ROS and RNS are necessary for the maturation process of cellular structures and play an important role in the host defense system. Indeed, phagocytes (neutrophils, macrophages, and monocytes) release free radicals to destroy invading pathogenic microbes as part of the body's defense mechanism against disease [8, 16]. The importance of ROS production by the immune system is clearly shown by patients with granulomatous disease. These patients have defective membrane-bound nicotinamide adenine dinucleotide phosphate (NADPH) oxidase production that makes them unable to produce the superoxide anion radical (O_2_^−^•), thereby resulting in multiple and persistent infections [8, 13]. Macrophages are activated when there is infection or inflammation, with toll-like receptors releasing NO or oxygen free radicals that may damage the tissue. Free radicals also induce proinflammatory cytokines [17]. Antioxidants are inhibitors of oxidation, either produced endogenously or received from exogenous sources. The role of antioxidants is to neutralize an excess of free radicals, to contribute to disease prevention, and to protect the cells against the toxic effects of oxidants, such as deoxyribonucleic acid (DNA) mutations and malignant transformations [1, 6]. It was reported that the use of antioxidants greatly enhances immune cell function, helping to control many bacterial and viral infections, reverse the imbalance between oxidants and antioxidants at the site of oxidant injury, and prevent progressive tissue damage [18]. Antioxidants are suggested for treatment of HIV, hepatitis C, Japanese encephalitis, and tuberculosis with anti-inflammatory features [18–21]. Currently known endogenous antioxidants include superoxide dismutase (SOD), catalase, glutathione peroxidase, glutathione reductase, peroxiredoxin, thioredoxin reductase, glutathione, flavonoids, bilirubin, uric acid, melatonin, thiols, reduced coenzyme Q, alpha-lipoic acid, endogenous organic selenium, and the metal-binding proteins transferrin, ferritin, lactoferrin, ceruloplasmin, and albumin. Exogenous antioxidants include vitamin C, vitamin E, carotenoids, stilbene antioxidants, phenolic acids, flavonoids, oil lecithins, acetylcysteine, exogenous selenium, zinc, magnesium, and copper [1, 2]. All of those molecules seem to be probable targets in the management of oxidative stress-induced diseases.

Oxidative stress, which occurs when there are more toxic free radicals produced than can be neutralized by antioxidant mechanisms, is an increasingly important topic among biological researchers. Under normal conditions, it is a continuing process of our bodies that begins before birth [22, 23]. ROS and RNS play dual roles as both toxic and beneficial compounds. The delicate balance between their two opposite effects is clearly an important aspect of life. At low or moderate levels, ROS and RNS exert beneficial effects on cellular responses and immune function. At high concentrations, they generate oxidative stress, a deleterious process that can damage cell structures such as DNA, lipids, and proteins [6]. Oxidative stress initiates structure modifications and function modulation in nucleic acids, lipids, and proteins. Of these, lipids are the most susceptible to oxidation. Oxidative degradation of lipids yields malondialdehyde, 4-hydroxynonenal, and isoprostanes, from unsaturated fatty acids. Protein damage may occur with thiol oxidation, carbonylation, side-chain oxidation, fragmentation, unfolding, and misfolding, resulting in loss of backbone and the side chain of proteins. ROS damage nucleic acids, and 8-hydroxydeoxyguanosine is an index of DNA damage [24]. Oxidative injury occurs when excessive production of ROS/RNS emerges and cannot be counteracted by the antioxidants. The imbalance between the oxidative and antioxidative systems may trigger some factors that cause oxidative damage in the cell. This leads to disease such as bacterial, viral, and parasitic infections, autoimmune disorders, malignancies, atherogenic activity, diabetes, kidney diseases, skin diseases, and neurodegeneration [1, 22, 25]. Moreover, bronchopulmonary dysplasia (BPD), retinopathy of prematurity (ROP), necrotizing enterocolitis (NEC), patent ductus arteriosus (PDA), periventricular leukomalacia (PVL), respiratory distress syndrome (RDS), intrauterine growth retardation (IUGR), and congenital malformation have also been reported to be oxidative stress-related neonatal diseases [20, 21, 23, 26–28].

## 2. Oxidative Stress-Related Disease in Preterm and Newborn Infants

**Figure 1:**
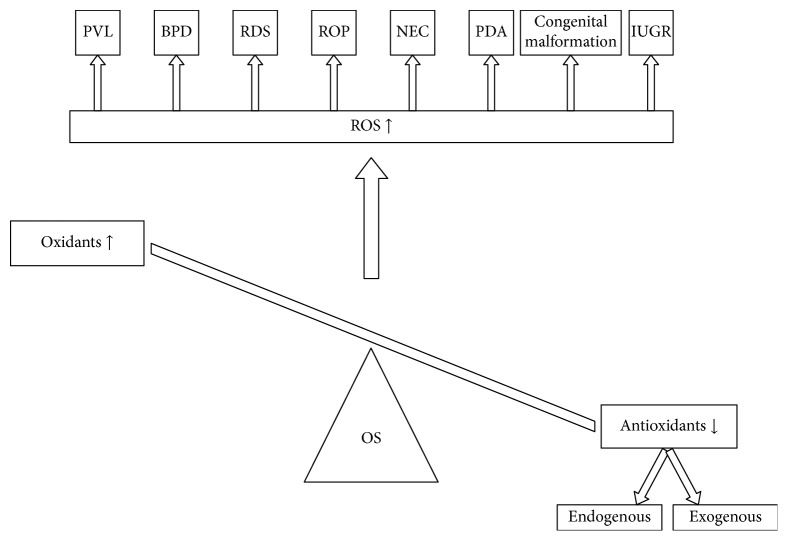
The imbalance between prooxidants and antioxidants in “oxygen radical disease of neonatology.” BPD, bronchopulmonary dysplasia; ROP, retinopathy of prematurity; NEC, necrotizing enterocolitis; PVL, periventricular leukomalacia; PDA, patent ductus arteriosus; RDS, respiratory distress syndrome; IUGR, intrauterine growth retardation; OS, oxidative stress; ROS, reactive oxygen species.

In 1988, Saugstad hypothesized that BPD, NEC, intracranial hemorrhage, PDA, and other possible diseases are not distinct, but all belong to one entity, “the oxygen radical disease of neonatology,” that has different symptoms according to which organs are mostly affected (Figure 1) [29]. Premature infants are especially susceptible to oxidative stress, and newborns are also susceptible for reasons including the following.

*(1) Hypoxic-Hyperoxic Challenge.* In the uterus, infants have a hypoxic environment, with 20–25 mmHg oxygen tension (PO_2_). However, they are born into an extrauterine normoxic environment of approximately 100 mmHg PO_2_ [30]. Some newborns require resuscitation with supplemental oxygen in the delivery room after being exposed to the hyperoxic environment. Rizzo et al. reported that increased oxygen tension induces elevated production of ROS in animal studies [31].

*(2) Infections.* Infants, especially preterm infants, are more susceptible to infections because infants are relatively immunodeficient [32].

*(3) Antioxidant Defense Deficiency.* Preterm infants and newborns have reduced antioxidant defense processes, including decreased levels of vitamin E, *β*-carotene, melatonin, ceruloplasmin, transferrin, and erythrocyte SOD [30]. Some antioxidants such as ascorbate and bilirubin are present in high concentration in newborns but only for a short time after birth [33].

*(4) High Levels of Free Iron.* Newborns have higher levels of free iron than older children, which cause an increased Fenton reaction, leading to the production of the highly toxic hydroxyl radical [17].

### 2.1. Periventricular Leukomalacia

In 2014, 1 of every 10 babies was born premature in the United States [34]. Every year, more than 20 million infants are born weighing less than 2.5 kg. Very-low-birth-weight infants, those infants born weighing less than 1.5 kg, are susceptible to adverse outcomes [35]. The incidence of PVL based on ultrasonographic findings ranges from 5% to 15% in very-low-birth-weight infants [36]. Neuropathologic evidence of PVL is found in 25% to 75% of very-low-birth-weight infants who die. PVL refers to injury to cerebral white matter that occurs in a characteristic distribution and consists of periventricular focal necrosis, with subsequent cystic formation, and more diffuse cerebral white matter injury [37].

Prevention of PVL will require new insights into its pathogenesis. The pathogenesis of this disease is related to the following factors:

(i) Incomplete development of the vascular supply in the cerebral white matter.
(ii) A maturation-dependent impairment in the regulation of cerebral blood flow underlying a propensity to ischemic injury to cerebral white matter.
(iii) Oligodendroglial precursor cells being the major cellular target of maturation-dependent vulnerability in PVL.
(iv) Maternal/fetal inflammation or infection causing oxidative stress.
(v) Elevation in extracellular glutamate causing toxicity to oligodendroglial precursors.

It has been shown that attacks by radicals, deficiency of antioxidant defenses, and active acquisition of iron derived from hemorrhage contribute to the pathological processes of disease during oligodendroglial differentiation. In consequence, deadly ROS and apoptotic oligodendroglial death may be the underlying reasons for PVL [38, 39].

### 2.2. Respiratory Distress Syndrome

More than half of extremely-low-birthweight (<1 kg) newborns will have some type of respiratory distress, and in that population RDS is the most common diagnosis (50.8%). The main factor for RDS is prematurity [40]. The pathophysiological factors include the following:

(i) Insufficient/dysfunctional surfactant resulting in collapsed alveoli, atelectasis, ventilation-perfusion mismatching, and subsequent hypoxemia and respiratory acidosis [40].
(ii) Sudden increase in oxygen supply after birth leading to an overproduction of ROS and depletion of antioxidants.
(iii) Hyperoxygenation destroying the vascular and alveoli endothelial cells.
(iv) Oxidant stress promoting expression of cytokines and the inflammatory process (interleukin-6, interleukin-8, and tumor necrosis factor-*α*) [41, 42].

### 2.3. Bronchopulmonary Dysplasia

Damage starts with the first postnatal breaths in lungs of premature infants. Infants weighing less than 1500 g at birth have BPD ranges between 15% and 50%, and ranges decrease by gestational age [43, 44]. The pathogenesis of BPD is complex. Some defined factors include the following:

(i) Reduced alveolar volume.
(ii) Deficiency of surfactant.
(iii) Immature extracellular matrix.
(iv) Inflammation unrelated to infection [43, 45].
(v) Oxygen free radicals produced after exposure to oxygen.
(vi) Oxidative stress activating inflammatory cells and increasing proinflammatory cytokines.
(vii) Oxidative stress causing injury to the respiratory tract epithelium and inactivating surfactant [44].
(viii) High tidal volume positive-pressure ventilation producing inflammation.
(ix) PDA with increased pulmonary blood flow triggering the inflammatory cascade and stimulating neutrophil margination and activation in the lung [43, 45].

### 2.4. Retinopathy of Prematurity

ROP, a proliferative retinopathy affecting premature infants, continues to be a leading cause of lifelong visual impairment among children in the developed countries. ROP causes visual loss in 1300 children and severe visual impairment in 500 children each year in the United States alone. The overall incidence of ROP is 0.17%, but it is nearly 16% for premature infants [46]. Basic research into the pathogenesis of ROP contributes to further understanding of retinal development, angiogenesis, and intraocular neovascularization [28, 46]. Currently known ROP pathogenetic factors after birth by hyperoxia include the following:

(i) Inhibition of retinal vascularization.
(ii) Loss of the nutrients and growth factors at the maternal-fetal interface.
(iii) Stopped blood vessel growth, with subsequent hypoxia because of retinal maturation and increasing metabolic demand.
(iv) The hypoxic retina stimulating expression of the oxygen-regulated factors that stimulate retinal neovascularization by using erythropoietin and vascular endothelial growth factor [47].
(v) Oxygen fluctuations inducing cells to produce NADPH oxidase, which causes increased ROS as well as apoptosis of endothelial cells, which contribute to avascular retina [38].

### 2.5. Necrotizing Enterocolitis

The incidence of NEC is approximately 1 per 1000 live births. For infants under 1500 g, the incidence increases to between 2.3% and 12%. Both the incidence and case fatality rate of NEC are inversely correlated with birth weight; about 30% of babies <1500 g with NEC will not survive [48, 49]. Pathogenetic factors of NEC include the following:

(i) Bacterial lipopolysaccharides increasing inducible nitroxide synthase activation in the enterocytes of neonates, triggering ROS production [41].
(ii) Multifactorial etiologies, including inflammation, ischemia, and cytokines (tumor necrosis factor-*α*, interleukin-6) producing a high level of free radicals [50, 51].
(iii) Local ischemia of the intestinal tissue and reperfusion triggering production of reactive species through some enzymes such as xanthine oxidase [50].
(iv) ROS and free radicals contributing to the disruption of the immature gut barrier [48, 51].
(v) Platelet-derived growth factor [52].

### 2.6. Patent Ductus Arteriosus

PDA, which has an incidence of 1 per 2000 in term neonates, is the persistence of the fetal communication between the descending aorta and left pulmonary artery. This congenital defect allows blood to bypass the fetal lungs and be directed into the descending aorta to supply structures below this region. It usually closes soon after birth under the physiologic effects of elevated oxygen level. Defects can range in size from so small as to be undetectable to large enough to cause volume loading of the left ventricle and pulmonary hypertension. PDA is seen more frequently (20% to 60%) in preterm infants [53], particularly those born at <30 weeks' gestation [54].

In 1971 Fay showed that there were oxygen sensors in the ductus arteriosus [55]. Further studies showed that the ductus arteriosus is affected by changes in PO_2_, with changes in the redox state producing ROS [56, 57]. Intrauterine hypoxia maintains the patency of the ductus arteriosus. However, at the time of birth, ROS increases when PO_2_ changes from fetal to neonatal levels [58]. In addition to this, hemodynamically significant PDA may cause hypoperfusion of organs, which can cause diseases such as NEC, BPD, and acute renal insufficiency [59, 60]. Hypoperfusion, ischemia, and chronic hypoxia lead to the production of oxygen radicals [61].

### 2.7. Congenital Malformation

Congenital anomalies are important causes of childhood death, chronic illness, and disability all over the world and they may have a significant impact on individuals, families, healthcare systems, and societies. It is estimated that 276,000 newborns die in the first month of life every year from congenital anomalies. The most common severe congenital anomalies are heart defects, neural tube defects, and trisomy 21. Although congenital anomalies may be genetic, infectious, nutritional, or environmental in origin, most often it is difficult to identify the exact causes [62]. It was stated in recent reports that there may be an association between oxidative stress and congenital malformations [63–65]. Oxidative stress has been defined as harmful radicals attacking biological molecules such as DNA, lipids, and proteins [66]. However, this relationship between oxidative stress and congenital malformation and the exact nature of the damage are not clear and need further investigation.

### 2.8. Intrauterine Growth Restriction

IUGR is a complication of pregnancy, often described when the fetus is estimated to be small for gestational age [67]. The reported incidence of IUGR ranges between 3% and 7%. IUGR is most probably a consequence of placental ischemia/hypoxia [68]. Some mechanisms involving IUGR and oxidative stress are as follows:

(i) High metabolic demand and elevated requirements for tissue oxygen in pregnancy [69].
(ii) Increased rate of production of ROS, oxidative stress, and lipid peroxidation compared with nonpregnant women.
(iii) Uncontrolled production of lipid peroxides resulting in additional oxidative stress [70].
(iv) Placental ischemia/hypoxia resulting in the release of products into the maternal circulation, which triggers preeclampsia as well as IUGR [71].
(v) Development during the late second or third trimester when the mother's antioxidant capacity to cope is limited.
(vi) Increased antioxidants such as vitamin E, ceruloplasmin, and erythrocyte thiols and increased iron-binding capacity; otherwise, serum iron concentrations progressively decrease.
(vii) Insufficient increase in antioxidants trying to counter the increase in oxidative stress and lipid peroxidation.

In conclusion, damage to cell integrity, cell membrane function, organelle membranes, and protein synthesis is a major cause of maternal and fetal morbidity [70].

## 3. Diagnosis of Oxidative Stress-Related Neonatal Disease

Currently it is known that oxidative stress is important in the pathogenesis of various kinds of neonatal disease, and there is a need for more information about and investigations of the manifestations and diagnosis of oxidative stress in neonates.

Giuffrè et al. [72] stated that glutathione, lipid hydroperoxides, and heat shock protein chaperonin 60 in the newborn's serum might have functional and diagnostic significance for oxidative stress. There are some studies of the diagnosis of oxidative stress-related neonatal disease in both humans and animals. Serum selenium is a constituent of the oxidant enzyme glutathione peroxidase and is vital for antioxidant defense [73]. El-Mazary et al. [74] showed that neonates with hypoxic-ischemic encephalopathy had lower serum selenium levels than normal healthy neonates. Mukhopadhyay et al. [75] reported that levels of antioxidants such as vitamin C and glutathione are reduced, and levels of serum malondialdehyde and protein carbonyl are different, in children with congenital malformation and healthy children. Therefore, these markers may be a target for studies that focus on the diagnosis of oxidative stress-related diseases [75]. Kumar et al. [76] claimed that increased levels of plasma and cerebrospinal fluid malondialdehyde are related to perinatal asphyxia.

To evaluate oxidative stress markers in neonates with IUGR, antioxidant enzyme (SOD, catalase, and glutathione peroxidase) activities and levels of antioxidants were measured. It was found that there were significantly lower levels of enzyme activities in the IUGR group [77] than in a control group. Cancelier et al. [78] demonstrated that thiobarbituric acid-reactive substances, which are an oxidative stress marker, were significantly higher in cord blood of infants with neonatal sepsis, and thiobarbituric acid-reactive substance levels were independently related to the development of neonatal sepsis. Lista et al. [79] noted that lung inflammatory response in preterm infants with RDS may be assessed by measuring proinflammatory cytokines in tracheobronchial aspirate fluid; if there is inflammation, lungs are more susceptible to oxidant stress. In 2015, Tataranno et al. [80] reported that discovery and validation of specific plasma oxidative stress markers of neonatal brain injuries give an idea of neonatal neuroprotection. According to the authors, prostanoids and nonprotein bound iron could be used as specific plasma oxidative biomarkers reflecting oxidative stress injury to neuronal cells. Eventually Marseglia et al. [81] concluded that visfatin could be a new marker of oxidative stress in preterm newborns. Visfatin is an adipocytokine involved in oxidative stress and an important mediator of inflammation that induces dose-dependent production of both proinflammatory and anti-inflammatory cytokines.

## 4. Therapeutic Approach to Oxidative Stress-Related Neonatal Disease

Neonates, particularly those born prematurely, have an incomplete detox response to free radicals. To passivate oxidative stress-related damage in newborns, many therapeutic strategies to promote antioxidant status in newborns have been proposed. Supplementation with enzymatic and/or nonenzymatic antioxidants has been experimented with, but the results were mixed [50]. It was reported that an antioxidant supply can prevent oxidant stress-related disease, support the immune systems of neonates, reduce stillbirths, and enhance neonatal vitality [41].

Antioxidant defense mechanisms include endogenous antioxidant enzymes such as SOD, catalase, and glutathione peroxidase and nonenzymatic compounds such as glutathione, proteins (ferritin, transferrin, ceruloplasmin, and even albumin), and uric acid, coenzyme Q, and lipoic acid, which are all low-molecular-weight scavengers. Vitamins C and E, carotenoids, and phenolics (flavonoids-flavonols) have been identified as the major exogenous antioxidants [1]. Many potential therapeutic antioxidants have already been investigated, particularly in diseases of newborns [38]. It was shown in some reports that oxidative stress-mediated intestinal injury was reduced by the addition of SOD, glutathione peroxidase, and N-acetylcysteine, which reduces concentrations of intestinal tissue tumor necrosis factor-*α* via its anti-inflammatory and antioxidant properties [82, 83]. In various studies, some therapies including hyperbaric oxygen, medical ozone, and enteral glutamine alone or in combination with arginine have shown favorable effects on NEC by modulating antioxidative defense mechanisms [84–86]. Surech et al. [87] concluded that intratracheal administration of recombinant human copper zinc SOD caused an improvement in the antioxidant activity of some enzymes in premature infants. Melatonin and its metabolites are strong antioxidants and they have important functions to prevent mutilation of crucial molecules by free radicals. It was demonstrated that melatonin reduces all aspects of the ensuing damage in the ischemia and subsequent reperfusion model of the heart, kidney, liver, intestine, and brain in cases of excessive ROS [50]. In 2004, Gitto et al. [42] showed that melatonin treatment can reduce the severity of RDS in preterm newborns by reducing inflammation. Additionally, it has been shown that melatonin can be used in the treatment of hypoxic-ischemic encephalopathy in newborns [88]. Melatonin for PVL has been studied in animal models, and agomelatine and melatonin seem to be likely neuroprotective agents for the prevention of PVL [38].

Resveratrol (a phytoalexin synthesized by some plants) and epicatechin (a green tea extract) are considered new treatment modalities for ROP. Resveratrol as a nitric oxide mechanism modulator and caffeic acid were investigated in the pathogenesis of retinal neovascularization and some effects beneficial for the prevention of ROP were found [89, 90]. Vitamin E, D-penicillamine, intratracheal recombinant human SOD, and allopurinol are used for the treatment and prevention of ROP [91]. Paraoxonase-3 has been identified as an antioxidant that is systemically upregulated in late gestation of human fetuses and some animals such as rats and sheep. According to the findings of the study designed by Belteki et al. [92], paraoxonase-3 may be a therapeutic candidate for preterm infants. For neonatal brain injury, there are possible agents such as vitamins C and E, inhibitors of nitric oxide synthase, allopurinol, erythropoietin, albumin, docosahexaenoic acid, deferoxamine, prostaglandin inhibitors, magnesium sulfate, N-acetylcysteine, melatonin, lutein, and omega-3 polyunsaturated fatty acid [93]. Endotracheal administration of recombinant human SOD, melatonin, and surfactant replacement can reduce the lung injury in preterm newborns receiving mechanical ventilation for RDS [94]. Exogenous antioxidants such as vitamins A and E and recombinant human SOD are considered able to prevent BPD [38].

Saugstad [17] stated that oxidative stress may be triggered or already occurs in prenatal life or before initiation of therapy. Currently there is no accurate single or combination antioxidant or anti-inflammatory agent that has been found and routinely used [17].

## 5. Future Directions

### 5.1. New Human Oxidative Stress Source: Wireless Local Area Networks

New studies have started to focus on the relationship between oxidative stress and wireless local area networks (WLANs). Nowadays, WLANs are everywhere, such as workplaces, homes, and public places. Scientists are investigating possible biological effects of exposure to WLAN signals because of the increased usage [95]. There are a few human studies in addition to animal studies. In animal investigations, it has been shown that the oxidative stress of organs such as the brain, liver, testes, ovary, kidney, and eye increases with exposure to WLANs, especially in pregnant or newborn animals [96, 97]. Human studies have generally focused on fertility [98].

### 5.2. Genetic Mutations

Gene mutations related to oxidative stress have been discovered. Tuxworth et al. [99] showed that the lack of CLN3 function leads to a failure to control the response to oxidative stress and this causes juvenile neuronal ceroid lipofuscinosis (also known as Batten disease), a disease characterized by neuronal degeneration. In one study, preterm infants born by cesarean delivery were compared with preterm infants born by vaginal delivery in terms of H_2_O_2_-induced oxidative DNA damage and repair capacity (residual DNA damage) in peripheral blood mononucleated cells [100]. The authors reported that preterm infants born by cesarean delivery repair oxidative DNA damage more slowly than preterm infants born by vaginal delivery. It is currently unknown how gene expression is affected; however, there are some hypotheses. Schlinzig et al. [101] observed significantly higher global DNA methylation in white blood cells of newborns delivered by cesarean section, and physiological hypoxemia during vaginal delivery was conducted to increase antioxidant defenses, whereas in the normoxemic planned cesarean section there might be a slower cell cycle, possibly favored by prenatal administration of corticosteroids to the mother, or there could be a different regulation of DNA repair enzymes [100]. Decordier et al. [102] found that H_2_O_2_ repair capacity and chromosome/genome mutations in newborns are different from those in adults. They found no genotype with a significant effect on DNA repair capacity for the introduction of chromosome/genome mutations by oxidative stress. However, maternal antioxidant supplementation during pregnancy is important for protecting newborns against oxidative DNA damage [102].

## 6. Conclusion

Neonatal tissues are especially sensitive to oxidative damage because of the rapidly growing nature of their tissues, which makes them vulnerable to the harmful effects of free radicals. However, there are still gaps in our knowledge of the potential role of oxidative injury in the pathogenesis of neonatal diseases. Moreover, there are few publications on validated roles of oxidative stress biomarkers and antioxidants and their protective roles in this field. New studies should more extensively investigate the diagnostic and therapeutic value of various oxidative stress biomarkers and antioxidants to reduce oxidative tissue injury to developing newborns.
